# Familial Skewed X Chromosome Inactivation in Adrenoleukodystrophy Manifesting Heterozygotes from a Chinese Pedigree

**DOI:** 10.1371/journal.pone.0057977

**Published:** 2013-03-01

**Authors:** Zhihong Wang, Aizhen Yan, Yuxiang Lin, Haihua Xie, Chunyan Zhou, Fenghua Lan

**Affiliations:** Research Center for Molecular Diagnosis of Genetic Diseases, Fuzhou General Hospital, Fuzhou, China; Pasteur Institute of Lille, France

## Abstract

**Background:**

X-linked adrenoleukodystrophy (X-ALD) is an inherited neurodegenerative disorder caused by mutations in the *ABCD1* gene. Approximately 20% of X-ALD female carriers may develop neurological symptoms. Skewed X chromosome inactivation (XCI) has been proposed to influence the manifestation of symptoms in X-ALD carriers, but data remain conflicting so far. We identified a three generation kindred, with five heterozygous females, including two manifesting carriers. XCI pattern and the *ABCD1* allele expression were assessed in order to determine if symptoms in X-ALD carriers could be related to skewed XCI and whether skewing within this family is more consistent with genetically influenced or completely random XCI.

**Results:**

We found a high frequency of skewing in this family. Four of five females had skewed XCI, including two manifesting carriers favoring the mutant allele, one asymptomatic carrier favoring the normal allele, and one female who was not an X-ALD carrier. Known causes of skewing, such as chromosomal abnormalities, selection against deleterious alleles, *XIST* promoter mutations, were not consistent with our results.

**Conclusions:**

Our data support that skewed XCI in favor of the mutant *ABCD1* allele would be associated with the manifestation of heterozygous symptoms. Furthermore, XCI skewing in this family is genetically influenced. However, the underlying mechanism remains to be substantiated by further experiments.

## Introduction

X-linked adrenoleukodystrophy (X-ALD, MIM 300100), the most common peroxisomal disorder, is an inherited neurodegenerative disease caused by mutations in the *ABCD1* gene, which maps to chromosome Xq28. It is biochemically characterized by accumulation of pathognomonic saturated very long chain fatty acids (VLCFAs) in body fluids and tissues. It has been demonstrated that X-ALD not only affects males, but also female carriers. Approximately 20% of X-ALD carriers develop symptoms like adrenomyeloneuropathy (AMN) in middle age or later [1], but there have been limited studies specifically addressing this issue.

Skewing of X chromosome inactivation (XCI) is a process in mammals in which one X in every XX female somatic cell is transcriptionally silenced, and the determination of the X chromosome to be inactivated is usually random. Females are expected to have two cell populations: one with the maternal and one with the paternal X as the active chromosome. Skewed XCI is arbitrarily defined as more than 80% of cells showing a preferential inactivation of the same X chromosome [2]. Extremely skewed XCI (≥95%) is rarely found in normal females [3], and females with extremely skewed XCI have been found to cluster in families [4]. This suggests that XCI may not be completely random, but can be genetically influenced. Genetic mechanisms that may lead to skewing include chromosomal abnormalities, mutations conveying a proliferative advantage or disadvantage to a cell, and mutations within the X inactivation centre (XIC), such as *XIST* promoter mutations. However, in most cases, the cause of skewing is unknown.

Unfavorable skewing of XCI, where the X chromosome carrying a mutant allele is the predominantly active X, has been found in manifesting carriers of several X-linked disorders [5]–[8]. However, for many X-linked disorders, a consistent relationship between the XCI pattern and clinical phenotype has been difficult to demonstrate, and X-ALD is no exception. It has been suggested that XCI contributes to the phenotypic variability in X-ALD carriers, but data remain conflicting so far [9]–[11].

Here, we describe a family in which two females expressed the AMN-like phenotype. Our objective was to determine if symptoms in X-ALD carriers could be related to skewed XCI and whether skewing within this family is consistent with genetically influenced XCI.

## Methods

### Ethics Statement

The protocols for this study were evaluated and approved by the Ethics Committee of Fuzhou General Hospital. Written informed consent was obtained from all participants in this study. The ethics committee approved this consent procedure.

### Participants

**Figure 1:**
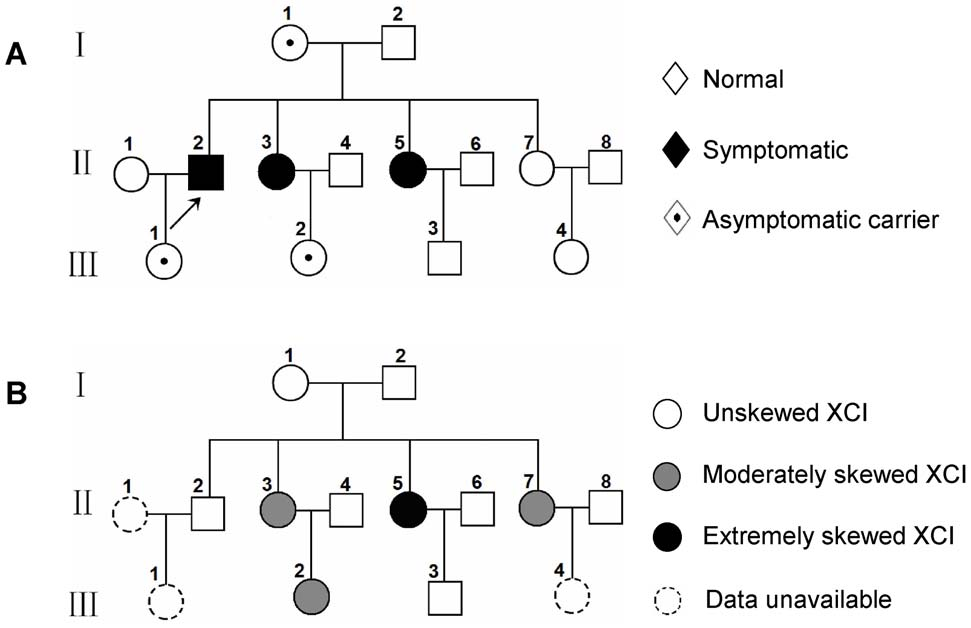
Pedigree analysis in the X-ALD phenotype (A) and XCI phenotype (B). A: Two of the five heterozygous females were clinically affected. B: XCI skewing was found in four female members, including extremely skewing in one symptomatic carrier. Moderately Skewed XCI: 80∶20≤XCI ratio<95∶5; Extremely Skewed XCI: XCI ratio≥95∶5.

The proband (Figure 1A, II2) was diagnosed with ALD-AMN by elevated VLCFAs in plasma at 34 years of age in Canada. Two of his sisters in mainland China, II3 and II5, 42 and 38 years old, respectively, suffered from progressive stiffness and weakness of their legs over the past 5 to 8 years. Their symptoms were not severe enough to require a walking aid. There were no sensory or sphincter disturbances or other neurological symptoms. Physical examination disclosed mild leg spasticity with normal strength. Their gaits were mildly spastic. The rest of neurological examination was normal. Brain and spinal MRI checks were normal. The proband had a 72-year-old mother (I1), another 36-year-old sister (II7) and a 21-year-old niece (III2), and neither of them had neurological symptoms.

### Biochemical Evaluation

VLCFAs were quantified in plasma by standard procedures.

### Mutation Identification

Peripheral blood was collected from the female participants of this family and genomic DNA was extracted with a DNA isolation kit (Qiagen Inc, Valencia, CA, USA). The entire *ABCD1* gene coding region, including flanking splicing sites, was amplified using appropriate primers. The purified PCR products were sequenced using an ABI 3730 Sequencer, and the mutation was characterized. Corresponding PCR products of normal controls (200 alleles) were also analyzed by restrictive digestion with Rse I to exclude the possibility of polymorphism. Pathogenicity of the mutation was assessed by in Silico analysis using three algorithms (PolyPhen, SIFT and PMut).

In addition, two carriers from two unrelated X-ALD families were found to have skewed XCI in favor of the wild-type *ABCD1* allele (data not shown), showing that the *ABCD1* allele-specific expression pattern mirrored the XCI pattern, probably because one allele of *ABCD1* was usually silenced as result of the XCI.

### X Chromosome Inactivation Analysis in Blood Leukocytes

XCI pattern was determined by quantitation of the methylation status of the polymorphic human androgen receptor (AR) locus according to Allen et al [12], with some modifications. Two DNA aliquots from each female participant were digested with restricted enzyme: one was HpaII, a methylation sensitive enzyme; and the other was the methylation insensitive enzyme, Dde I, which was used as a control for input DNA. Male DNA was used as the control for complete digestion. After digestion at 37°C overnight, the digested DNA was used to amplify a polymorphic CAG repeat within the AR gene with FAM-labeled fluorescent primers. The two AR alleles were then sized and quantified using an ABI 3100 Genotyper. The size of the allele indicates the parental origin and the area under the peak indicates the degree of amplification of the allele. The ratio of active to inactive AR alleles is a measure of the degree of XCI. It is defined as skewed when the ratio of peaks differs significantly from 50∶50, generally by more than 80∶20.

Besides the presented family, we also analyzed blood samples from 25 asymptomatic carriers and 10 noncarriers from 19 unrelated X-ALD families and 50 age-matched healthy female controls. The females who were uninformative at the AR locus were not included in the analysis.

### Transcript Analysis in Blood Leukocytes

Because genes are transcribed only from the active X chromosome, RNA transcripts were analyzed to investigate whether the mutant or the wild-type *ABCD1* allele was predominantly active. Total RNA was isolated from the peripheral blood of the female carriers with an RNA isolation kit (Qiagen Inc, Valencia, CA, USA), and 800 ng of total RNA was reverse transcribed with reverse transcriptase (Invitrogen Inc, Carlsbad, CA, USA). A fragment of 700 bp spanning the mutation was amplified with the forward primer: 5′-ACTGGCCCTGTCGTTCCG-3′, and the reverse primer: 5′-GTTGCGGGCAATAGTGAAG-3′, and the PCR product was then sequenced. T cloning of the corresponding PCR product was also performed and a total of 25 colonies were picked, from which recombinant plasmids with inserted PCR product were isolated and sequenced.

### *XIST* Genetic Analysis

PCR primers were used to amplify the minimal promoter of *XIST* in female participants of the family as described [13], and the PCR product was sequenced directly.

### Cytogenetic Studies

Cytogenetic analyses were performed by standard methods on PHA-stimulated peripheral blood lymphocytes. Bands were visualized at a resolution of 400–550.

## Results

### Plasma VLCFAs Level

Plasma VLCFAs assay showed the amount of C26:0, the ratios of C24/C22 and C26/C22 were higher than normal in I 1, II3, II5 and III2 (Table 1).

**Table 1:** Biochemical findings^a^.

Participants	C26^b^	C24/C22	C26/C22
II5	2.300	1.259	0.075
II3	2.222	1.252	0.037
II7	0.593	0.974	0.013
I 1	2.483	1.205	0.024
III2	2.375	1.425	0.042

a VLCFAs normal ranges: C26 0.378–0.642 µmol/L; C24/C22 0.606–1.16; C26/C22 0.011–0.023.

b unit: µmol/L.

### *ABCD1* Gene Mutation Analysis at Genomic DNA Level

**Figure 2:**
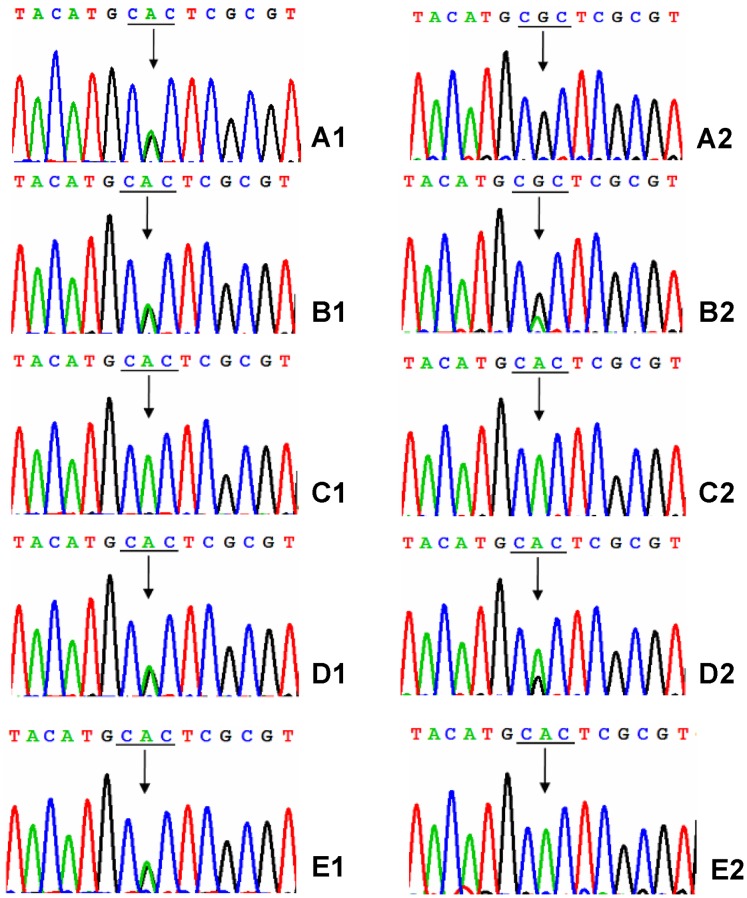
Sequence analysis in genomic DNA and cDNA of the female members. Genomic DNA sequencing showed a heterozygous pattern of the A-to-G-transition in II5, II3, I 1 and III2 (A1, B1, D1 and E1), but not in II7 (C1). cDNA sequencing showed solely or mainly the mutant allele to be expressed in II5 and II3 (A2 and B2), while solely or mainly the wild-type allele to be expressed in III2 and I 1 (E2 and D2). The position of codon is underlined and the arrow indicates the mutated base.

Sequence analysis of the PCR products of the female participants revealed a heterozygous mutation in I 1, II3, II5 and III2, but not in II7 (Figure 2, A1–E1). The mutation was in exon 1 of the *ABCD1* gene, at codon 283 (CAC→CGC), which resulted in the replacement of histidine by arginine (p.His283Arg). The mutation was not detected in 200 alleles of normal controls by restriction analysis with Rse I (data not shown). There was no other mutation detected in the *ABCD1* gene coding region of this family.

### X Chromosome Inactivation Analysis

**Figure 3:**
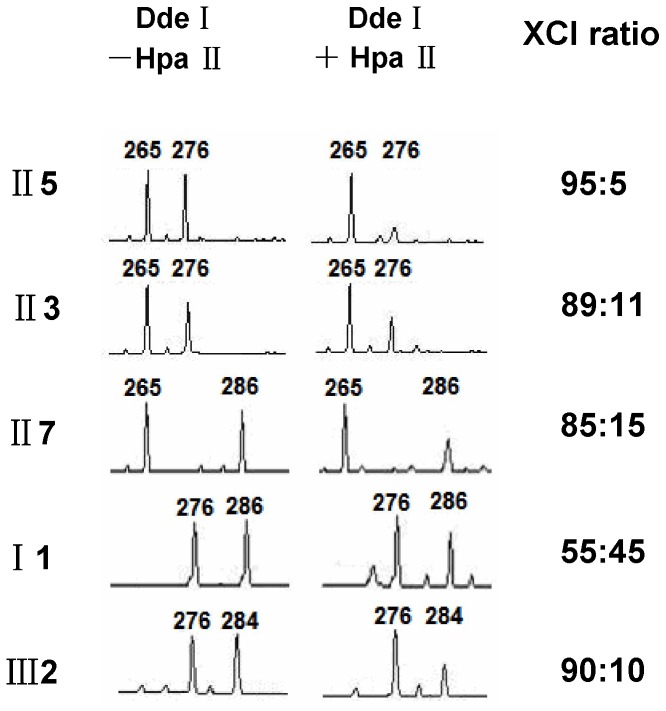
X chromosome inactivation analysis. After digestion with or without Hpa II, DNA was used to amplify a polymorphic CAG repeat within the androgen receptor (AR) gene. The size of the allele is determined by the number of repeats. The area under the peak indicates the degree of amplification of that allele. II5 and II3 inherited the allele 276 from their mother (I 1) and allele 265 from their father, II7 inherited the other allele 286 from I 1. After digestion with Hpa II, their paternal allele was predominantly amplified and represented the inactive. In contrast, III2 inherited the allele 276 from her mother (II3), which was predominantly amplified and represented the inactive. Thus four of them showed skewing of XCI (XCI ratio≥80∶20).

Digestion with Dde I (Figure 3, left column) showed the manifesting carriers II3 and II5 inherited a 265 bp AR allele from their father and 276 bp allele from their mother (I 1), while their unaffected sister II7 inherited the other 286 bp allele from their mother (I 1), and carrier III2 inherited a 276 bp allele from her mother (II3) and 284 bp allele from her father. Digestion with Hpa II (Figure 3, right column) showed that most of the inactive X chromosomes were of paternal origin in II3 and II5, while the maternal X chromosomes which carried the mutation, were predominantly active. In contrast, the maternal X chromosome of III2 carrying the mutation was predominantly inactive. The ratio of maternal to paternal allele expression was 95∶5, 89∶11, 85∶15 and 10∶90 in II5, II3, II7 and III2, respectively, indicating a high frequency of skewed XCI in the family (Figure 1B). These data also showed that skewed XCI in X-ALD carriers can occur in favor of the mutant or the wild-type allele.

XCI patterns of X-ALD carriers, related noncarriers and normal controls were summarized in Table 2. Five of 25 carriers (20%) demonstrated skewed XCI, including 1 carrier (4%) extremely skewing. Extremely skewing was not observed in related noncarriers and normal controls. The difference between the occurrence of XCI skewing in heterozygotes (20%) and controls (14%) was not significant (p>0.05; Fisher’s exact test).

**Table 2:** XCI pattern of X-ALD carriers, related noncarriers and normal controls.

XCI pattern	No. of carriers		No. of related noncarriers	No. of normal female controls
	asymptomatic	symptomatic		
Random XCI	20	0	8	43
Moderately Skewed XCI (80∶20≤XCI ratio<95∶5)	3	1	2	7
Extremely Skewed XCI (XCI ratio≥95∶5)	0	1	0	0
Total number	23	2	10	50

### Transcript Analysis in Leukocytes of Carriers

Sequencing chromograms (Figure 2, A2–E2) and T cloning (Table 3) of RT-PCR products showed solely or mainly the mutant *ABCD1* allele in manifesting carriers II5 and II3, respectively, indicating that the mutant allele was predominantly expressed. In contrast, the wild-type allele was predominantly expressed in asymptomatic carrier III2.

**Table 3:** T cloning findings.

Participants	No. of picked colonies	No. of mutant colonies	No. of wild-type colonies	Ratio of mutant to wild-type colonies
II5	25	25	0	100∶0
II3	25	20	5	80∶20
II7	25	0	25	0∶100
I1	25	9	16	36∶64
III2	25	0	25	0∶100

### Karyotype and *XIST* Mutation Analysis

The standard cytogenetic analyses showed the extremely skewed manifesting female (II5) had a normal karyotype. The sequence analysis of the *XIST* minimal promoter did not reveal any abnormalities of the amplified fragment (data not shown).

## Discussion

**Figure 4:**
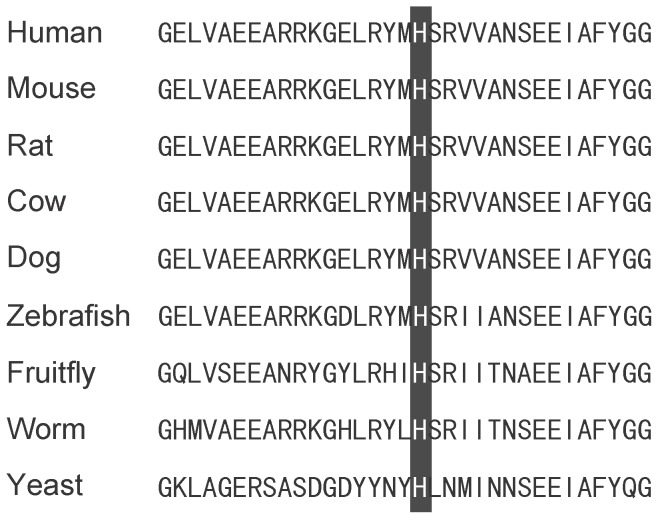
Alignment of ALDP proteins in different species. The black bar indicates the position of p.283H in the ALDP sequence.

In the family presented here, a missense mutation (p.His283Arg) was identified, which has not been reported in X-ALD database or other published data, indicating that it is a novel mutation in the *ABCD1* gene. For three reasons, it is unlikely a polymorphism or rare variant. First, the mutation was not detected in 200 alleles of normal controls. Second, the histidine at codon 283 is conserved among multiple species (Figure 4). Third, the in silico analysis of pathogenicity was performed using three algorithms (PolyPhen, SIFT and PMut), and all three prediction results revealed that p.His283Arg was classified as pathogenic. The unusual feature of this family is that there are two sisters affected with ALD-AMN, and both of them are heterozygous for this mutation. Having ruled out the possibility that they are compound heterozygotes, we searched for an alternative explanation for their AMN-like phenotype.

While it is often stated that in recessive X-linked disorders heterozygotes are asymptomatic, it is known that to some X-linked disorders the dichotomy between recessive and dominant does not apply and there are conditions where heterozygosity may represent a disease state. Besides X-ALD, these may include haemophilia A, myotubular myopathy, Duchenne muscular dystrophy and others [5]–[8]. In many cases, this has been shown to be due to unfavorable skewing of XCI. Carriers may therefore have a phenotype which varies from normal to affected, possibly depending on the degree of mosaicism.

Is skewed XCI responsible for ALD phenotype in female carriers? Conflicting results have been reported by different authors: Maier et al [9] stated that the manifestation of symptoms in X-ALD carriers was related to skewed XCI, and there was a significant correlation between the extent of skewing and the severity of neurological abnormalities; whereas Watkiss et al [10] and Salsano et al [11] failed to find any association between the neurological manifestations and XCI pattern. The discrepancies between these results might be attributable to differences in methodology, patient age and sample size. We had defined two distinct events segregating in the present family: the *ABCD1* mutation on the maternal X chromosome and the skewed pattern of XCI that prevents the expression of the wild-type *ABCD1* allele. We propose that the convergence of these is related with the clinical manifestations in these two heterozygous females, which is in agreement with the previous data of Maier et al.

In this family, five females from three consecutive generations were assessed for the *ABCD1* gene transcription and XCI pattern in blood leukocytes. Interestingly, four of five females in our study had skewed XCI, including two symptomatic sisters (II5 and II3) favoring the mutant allele, one asymptomatic carrier (III2) favoring the normal allele, and one female (II7) who was not an X-ALD carrier. This distribution suggested a genetic influence over XCI in this family.

We have ruled out chromosomal abnormalities as a mechanism of XCI skewing in a symptomatic carrier (II5). We have not formally determined the karyotypes of the other family members with skewed XCI. However, it is unlikely that their skewed XCI is due to an X-autosome translocation, as none have the completely skewed XCI expected in these translocations. Further, it is most likely that all of the skewed XCI in this family is due to a common mechanism. Therefore, we are not pursuing X-autosome translocations as a mechanism in any of the family members.

Some X-linked gene mutations conveying a proliferative advantage or disadvantage to a cell might lead to XCI skewing. However, it is unlikely that XCI skewing is related to the inheritance of the *ABCD1* mutation, as transcript analysis showed that skewing can be associated both with predominance of the mutant and the wild-type *ABCD1* allele. This observation does not go together with the reports of Salsano et al [11] and Migeon et al [14] suggesting that *ABCD1* mutations confer a proliferative advantage leading to XCI skewing in favor of the X chromosome with the mutation, however, this phenomenon could not be explained. In addition, there was no other known disease mutation segregating in this family to predispose them to unbalanced XCI.

Transcription of *XIST* is essential for X chromosome inactivation. The *XIST* gene has been fully sequenced, but its functional domains have not yet been fully identified. Mutations in the promoter of the *XIST* gene have been reported to cause familiar non-random XCI [13], [15]. However, neither the previously reported C43G mutation nor any other mutation within the minimal promoter was found in this family. Of interest, Pereira and Zatz [16] also did not find the C43G promoter mutation in 66 females with extremely skewed XCI, so it seems not to be a common cause of skewing.

In conclusion, our data support that skewed XCI in favor of the mutant *ABCD1* allele would be associated with the manifestation of heterozygous symptoms. Furthermore, XCI skewing in this family is genetically influenced. However, there is no obvious explanation for the XCI skewing in our case. The underlying mechanism triggering this phenomenon remains to be substantiated by further experiments. One could propose that there is an influence of a yet unidentified X-linked or autosomal locus and modifying factors affecting the mechanism.

Our finding implies that the demonstration of skewed XCI in a carrier does not necessarily result in a clinically detrimental influence. However, the finding of unfavorable skewed XCI in a carrier would be a valuable parameter for early identification of the carriers with a high risk of becoming symptomatic and for whom a possible preventive therapy would be warranted. Moreover, an analysis of XCI over time can be useful in the assessment of females with atypical presentations of ALD. Therefore, the possible role that XCI pattern plays in clinical manifestations of ALD carriers should be told in ALD-related counseling, while mentioning that the techniques for XCI and the *ABCD1* allele-specific expression pattern analysis is available in the laboratory.
